# Evaluation of the Performance of 18F-Fluorothymidine Positron Emission Tomography/Computed Tomography (18F-FLT-PET/CT) in Metastatic Brain Lesions

**DOI:** 10.3390/diagnostics9010017

**Published:** 2019-01-26

**Authors:** Alexandra Nikaki, Vassilios Papadopoulos, Varvara Valotassiou, Roxani Efthymiadou, George Angelidis, Ioannis Tsougos, Vassilios Prassopoulos, Panagiotis Georgoulias

**Affiliations:** 1 Department of Clinical Physiology and Isotope, Kanta-Häme Central Hospital, 20 Ahvenistontie Str., 13530 Hameenlinna, Finland; 2 Nuclear Medicine Department, University Hospital of Larissa, Mezourlo, 41110 Larissa, Thessaly, Greece; vvalotasiou@gmail.com (V.V.); gangel217@gmail.com (G.A.); 3 PET/CT Department, Hygeia Hospital, 4 Erythrou Stavrou Str., 15123 Athens, Greece; r.efthimiadi@hygeia.gr (R.E.); VPrasopoulos@hygeia.gr (V.P.); 4 Department of Hematology, Papageorgiou General Hospital, Perifereiaki Odos N. Efkarpias, 56429 Thessaloniki, Greece; vassiliospap@gmail.com; 5 Radiology Department, Hygeia Hospital, 4 Erythrou Stavrou Str., 15123 Athens, Greece; 6 Medical Physics Department, Faculty of Medicine, University of Thessaly, University Hospital of Larissa, Mezourlo, 41110 Larissa, Thessaly, Greece; tsougos@med.uth.gr; 7 Department of Nuclear Medicine, Hygeia Hospital-Mitera, 4 Erythrou Stavrou Str., 15123 Athens, Greece; 8 Nuclear Medicine Department, Faculty of Medicine, University of Thessaly, University Hospital of Larissa, Mezourlo, 41110 Larissa, Thessaly, Greece; pgeorgoul@uth.gr

**Keywords:** 18F-fluorothymidine positron emission tomography/computed tomography, brain metastases, FLT, PET/CT

## Abstract

18F-fluorothymidine (18F-FLT) is a radiolabeled thymidine analog that has been reported to help monitor tumor proliferation and has been studied in primary brain tumors; however, knowledge about 18F-FLT positron emission tomography/computed tomography (PET/CT) in metastatic brain lesions is limited. The purpose of this study is to evaluate the performance of 18F-FLT-PET/CT in metastatic brain lesions. A total of 20 PET/CT examinations (33 lesions) were included in the study. Semiquantitative analysis was performed: standard uptake value (SUV) with the utilization of SUVmax, tumor-to-background ratio (T/B), SUVpeak, SUV1cm^3^, SUV0.5cm^3^, SUV50%, SUV75%, PV50% (volume × SUV50%), and PV75% (volume × SUV75%) were calculated. Sensitivity, specificity, and accuracy for each parameter were calculated. Optimal cutoff values for each parameter were obtained. Using a receiver operating characteristic (ROC) curve analysis, the optimal cutoff values of SUVmax, T/B, and SUVpeak for discriminating active from non-active lesions were found to be 0.615, 4.21, and 0.425, respectively. In an ROC curve analysis, the area under the curve (AUC) is higher for SUVmax (*p*-value 0.017) compared to the rest of the parameters, while using optimal cutoff T/B shows the highest sensitivity and accuracy. PVs (proliferation × volumes) did not show any significance in discriminating positive from negative lesions. 18F-FLT-PET/CT can detect active metastatic brain lesions and may be used as a complementary tool. Further investigation should be performed.

## 1. Introduction

Since its introduction, positron emission tomography (PET) fused with computed tomography (CT) (PET/CT) has gained a crucial role in everyday clinical practice of medical imaging, particularly in oncology for both solid tumors and lymphomas. 18F-fluorodeoxyglucose (18F-FDG) has long been the radiopharmaceutical of choice for the PET/CT evaluation of oncology patients. However, it is less optimal for evaluation of intracranial lesions because of its high brain accumulation [1]. On the other hand, accurate diagnosis is of great importance for therapeutic guidance and prognostic estimation, considering the individualized approach in patient treatment.

Magnetic resonance imaging (MRI) is a useful tool for brain imaging of patients with neurological symptoms and is considered the imaging modality of choice for evaluation of intracranial lesions. However, several limitations restrict its utilization in all patients, such as pacemakers, while other conditions interfere with the interpretation of its results, such as post-therapy pseudoprogression and pseudoregression of primary brain tumors [2]. Advanced MR innovations, such as spectroscopy (MRS), diffusion, and perfusion techniques serve as auxiliaries to better understand and estimate abnormal brain lesions and to further guide patients’ diagnosis, treatment, and prognosis [2]. The reported sensitivity and specificity for MRS in brain tumors are 80.05% (95% CI = 75.97%–83.59%) and 78.46% (95% CI: 73.40%–82.78%), respectively [3]. 

In the spectrum of PET imaging, several radiopharmaceuticals have been developed and are currently utilized for PET brain imaging, including 18F-FDG, 11C-choline (11C-CH), 18F-choline (18F-CH), 18F-fluoromisonidazole (18F-FMISO), radiolabeled amino acids such as 11C-methionine (11C-MET), 18F-fluoroethyltyrosine (18F-FET), and 18F-fluorodopa (18F-FDOPA). Each tracer reflects different pathophysiological characteristics, providing a more individualized approach in the clinical setting. In brain cells, amino acids contribute to protein synthesis, neuroactive peptide synthesis, metabolism, and synaptic transmission [1]. Choline is a substrate of phospholipids and cell membranes [4], while fluoromisonidazole serves as a marker of hypoxia [5]. 

18F-fluorothymidine (18F-FLT) is a radiolabeled thymidine analog considered to reflect tumor proliferation. After passive diffusion and/or active transportation, 18F-FLT enters the cell, where it is phosphorylated by thymidine kinase-1 (TK1), and remains trapped intracellularly [6,7]. For brain tumor imaging, disruption of the blood-brain barrier (BBB) is an important step for 18F-FLT uptake [8,9]. 18F-FLT-PET/CT imaging has been used in investigating both initial presentation and recurrence of gliomas. 18F-FLT uptake is reported to discriminate between high and low grade gliomas with a sensitivity of ~92% in newly diagnosed brain tumors [10]. Moreover, tumor-to-background (T/B) ratio has also been reported to correlate with tumor grade in newly-diagnosed and recurrent brain lesions [11,12]. Finally, 18F-FLT uptake has been found to correlate with tumor proliferation values (i.e., Ki-67), yielding prognostic information regarding progression-free and overall survival [13,14]. 

Although 18F-FLT has been evaluated for primary brain tumors, only scarce information exists for its use in the investigation of metastatic tumors. In this retrospective study, we examined the potential role of 18F-FLT-PET/CT in metastatic brain lesions. The main objective of the study was to assess the sensitivity, specificity, and accuracy of 18F-FLT-PET/CT in detecting metastatic brain lesions regardless of prior treatment, with the utilization of semiquantitative approach. Secondary objectives were to assess whether there was an optimal cutoff in semiquantitative parameters that could better separate active from non-active tumors.

## 2. Materials and Methods

### 2.1. Patients

Data from consecutive patients, who underwent 18F-FLT-PET/CT in our department from October 2011 until June 2015 for metastatic brain lesions, were collected. In total, 20 18F-FLT-PET/CT examinations were performed in 14 patients. The primary site was the lung in 9 PET cases, breast in 7 PET cases, melanoma in 2 PET cases, and thymoma in 2 PET cases. All patients had undergone treatment at some point of the course of their disease according to protocols (chemotherapy, radiotherapy, surgical excision). There were three patients who had never undergone brain radiotherapy, while in one case the patient had undergone surgical excision after radiotherapy. Per examination analysis refers to the number of 18F-FLT-PET/CT examinations. In all cases patients had undergone MRI (pre and post gadolinium (Gd) T1-weighted, T2-weighted and Flair) within 2 months of 18F-FLT-PET/CT examination (in 17/20 cases within a month). All patients were informed about the radiation protection rules. Written informed consent for undergoing FLT-PET/CT examination was also obtained. The study protocol was accepted by the scientific board of Hygeia hospital. Patients’ characteristics are summarized in Table 1.

### 2.2. 18F-FLT-PET/CT

18F-FLT was synthesized in Biokosmos (Lavrio, Greece) and distributed to our hospital. PET/CT was performed 55 ± 10 min after the intravenous administration 18F-FLT (310–420 MBq) on a Siemens Biograph LSO 16 slices PET/CT device by Siemens. Patients fasted for at least 4 h prior to the administration of the radiopharmaceutical. A 10 min imaging of the brain was received. Low-dose CT was acquired just before the PET examination using a 16-slice helical CT scanner. Data was collected in a frame mode and analyzed at a Siemens multimodality workplace (MMWP). Results were analyzed on a per-lesion basis. PET/CT images were interpreted by a nuclear medicine physician and a radiologist. For the semiquantitative analysis, the following parameters and lesion-proliferative indices and volumes were used: SUVmax, tumor-to-background ratio (T/B: SUVmax of the tumor/SUVmean of the normal brain background), SUVpeak, SUV1cm^3^ (SUV average obtained using 1 cm^3^ around SUVmax), SUV0.5cm^3^ (SUV average using 0.5 cm^3^ around SUVmax), SUV50% (SUV average obtained from volumes having SUV > 50% of SUVmax), SUV75% (SUV average obtained from volumes having SUV > 75% of SUVmax), proliferation multiplied by volumes using SUV50% and SUV75% (PV50% and PV75%, respectively, volume × SUV50% and volume × SUV75%). SUVmax of the lesions was derived by manually placing a volume of interest (VOI) around the lesion. For the T/B calculations, a VOI was manually drawn on the opposite normal encephalic/cerebellar hemisphere and SUVmean was obtained. In cases of multiple lesions, the background activity was measured at either hemisphere, avoiding areas with increased 18F-FLT uptake. In total, 33 lesions were evaluated. The final diagnosis was established by clinical and MRI follow-up, and, in one case, by biopsy results. Lesions were characterized as positive or negative according to the final diagnosis.

### 2.3. Statistical Analysis

Primary outcome was the evaluation of sensitivity, specificity, and accuracy of the 18F-FLT-PET/CT in detecting metastatic brain lesions. First, the lesions were classified into two groups according to the final diagnosis: positive or negative. Subsequently, for each semi-quantitative parameter (SUVmax, T/B, SUVpeak, SUV1cm^3^, SUV0.5cm^3^, SUV50%, SUV75%, PV50%, and PV75%), median values and interquartile range (IQR), as well as mean and standard deviation (SD) were calculated for each group; comparisons were made using the Kruskall-Wallis test and independent *t*-test, respectively. *p*-values < 0.05 were considered statistically significant.

For the parameters that were significantly different between the two groups, a receiver operating characteristic (ROC) curve analysis was performed. The discriminatory potential of each of these parameters were assessed using the area under the curve (AUC) value. The optimal cut-off point for each parameter (optimal diagnostic point) was defined as the point on the curve that is closest to the top of the y-axis, so that the combination of sensitivity and specificity can be maximized [15].

## 3. Results

**Figure 1:**
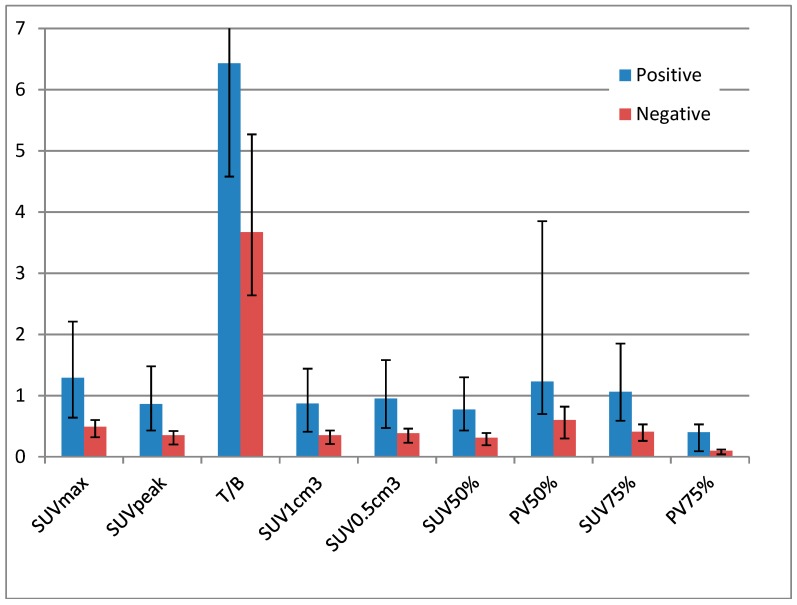
Median and IQR depicted as graph.

In total, 33 lesions in 20 18F-FLT-PET/CT examinations were analyzed: in 15 PET/CT cases there was 1 lesion, in 1 PET/CT case there were 2 lesions, in 2 PET/CT cases there were 3 lesions, in 1 PET/CT case there were 5 lesions. In one case there were three older lesions and at least four tiny lesions in MRI with Gd enhancement and without edema, one of which close to a larger one. PET/CT clearly visualized the four lesions, one had low uptake (SUVmax 0.42 and considered negative according to SUVmax cutoff) and did not visualize the last one. Mean (± standard deviation (SD)) values of SUVmax, T/B, SUVpeak, SUV1cm^3^, SUV0.5cm^3^, SUV50%, SUV75%, PV50%, and PV75% for each group are shown in Table 2. The corresponding median values and IQR are shown in Table 3 and Figure 1. The difference between positive and negative lesions was statistically significant when compared using SUV values, but not for the PV50% and PV75%.

**Figure 2:**
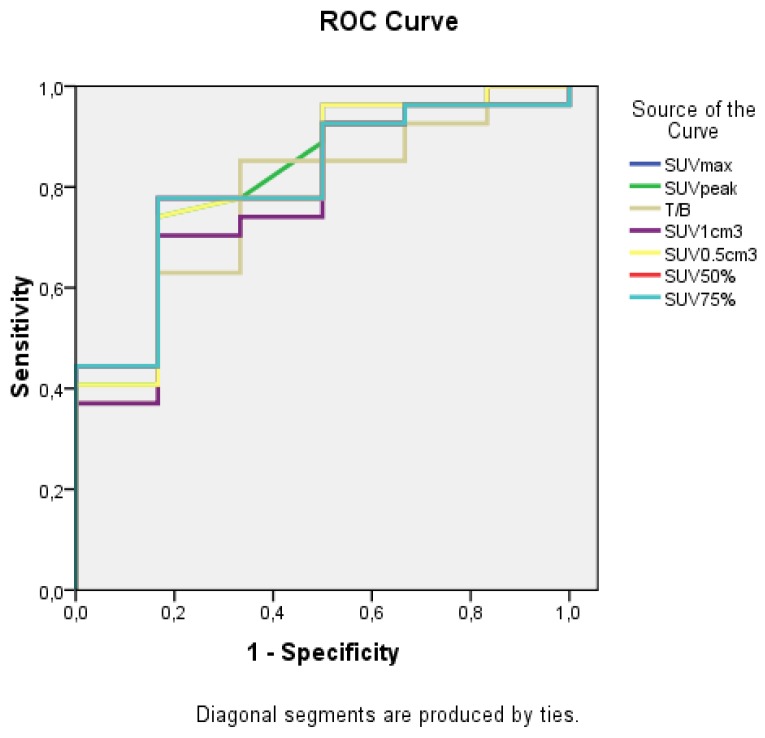
ROC curve for the parameters that differ significantly between negative and positive lesions. The curves are close to one another, but SUVmax has the best performance.

**Figure 3:**
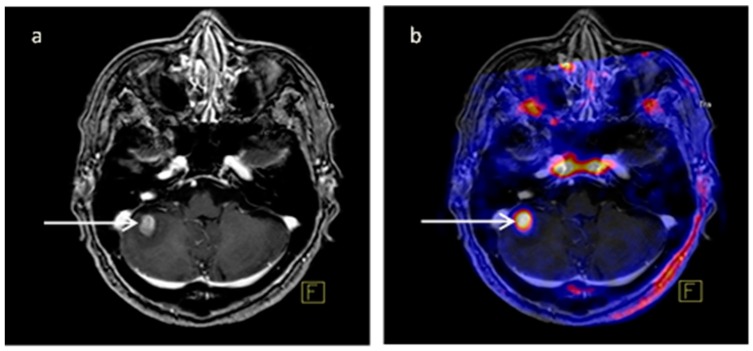
MRI-T1 post gadolinium sequence (**a**) and fused PET/MRI (**b**) images. Cerebellar metastatic lesion (arrows) 4 months after radiotherapy. Primary cancer site is breast. SUVmax, T/B, SUVpeak 2.62, 13.1 and 1.54 respectively. PV50% and PV75% 1.302 and 0,401 respectively.

Using ROC curve analysis, the optimal cutoff values of SUVmax, T/B, and SUVpeak for discriminating active from non-active lesions were 0.615, 4.21, and 0.425, respectively. Additionally, AUC parameters and a 95% Confidence Interval (95%CI) are shown in Table 4 and Figure 2; false results, sensitivity, specificity, and accuracy for each value according to optimal cutoff are shown in Table 5. In ROC curve analysis, AUC (95%CI) is higher for SUVmax (*p*-value 0.017), while using optimal cutoff T/B shows the highest sensitivity and accuracy. Using SUVmax there was one false positive examination: the patient had undergone surgical excision of brain metastatic melanoma and radiotherapy; follow up MRI ~2 years after the therapy showed an increase of Gd enhancement which pointed to recurrence of disease and so the patient underwent 18F-FLT-PET/CT which revealed avidity in the lesion; 19 days after the PET examination, the patient had a sample biopsy which was negative and therefore the lesion was considered negative; finally the patient underwent g-knife 8.5 months after biopsy for recurrent disease. Using T/B there was another FP result: in this case the SUVmax was low, however due to low background activity the T/B was positive using cutoff values. Figure 3 shows a brain metastatic lesion; MRI and fused PET with MRI images are shown; the corresponding values are mentioned in the caption.

## 4. Discussion

Metastatic brain tumors consist of a heterogeneous group of tumors with variable characteristics depending mostly on the primary tumor. Breast, lung, and melanoma are usual primary tumors that metastasize to the central nervous system. Moreover, brain metastases usually coexist with metastatic sites in other organs of the same patient and usually the patients have received multiple treatments, including surgeries, chemotherapies, radiotherapy, and novel targeted treatments with variable results. The accumulation of 18F-FLT in brain metastatic lesions could be a combination of BBB breakdown and the aggressiveness, vascularization, and other molecular characteristics of the metastatic tumor, as well as of the specific molecular characteristics of the primary tumor. Moreover, 18F-FLT uptake could also depend on the environmental and molecular characteristics of the site where the primary tumor metastasizes, as well as on the applied treatments. 

As a marker of proliferation, FLT seems to be useful in further evaluating the activity of metastatic brain lesions. O’Sullivan et al. reported that there was quite a good correlation between FLT and MRI in assessing treatment responses using a novel agent for breast cancer derived brain metastatic disease and demonstrated the supporting valuable contribution of FLT-PET in these cases [16]. Compared to FDG in head and neck cancer and thoracic malignancies, FLT seems to perform better in metastatic intracranial lesions [17,18]. Ongoing trial evaluate FLT-PET performance in treatment response and resistance prediction in brain lesion of melanoma patients [19]. In this study, using different semiquantitative measurements and proliferative indices, we explored the performance of 18F-FLT in metastatic brain lesions. In general, sensitivity in metastatic intracranial foci detection is high in our presented cases. Possible false negative results could be due to less BBB disruption or to the size and location of the metastatic lesion. False positive results could be related to prior treatments and BBB disruption.

SUVmax is the most common semiquantitative method used in everyday clinical practice. Generally, SUVmax has been found to correlate with tumors’ aggressiveness and response to treatments, while SUVmax thresholds have been used for discriminating between benign from malignant diseases, and in the case of gliomas, between low and high grades. Because of low SUVmax values for 18F-FLT brain PET measurements, we also used T/B and other SUV values and proliferative volumes for our analysis [20,21,22,23,24]. To our knowledge, this is the first study with such an approach for metastatic brain lesions with 18F-FLT.

PV50% and PV75% values were not found to be significant in discriminating positive from negative lesions (*p* value > 0.05). A possible explanation is that these values are a mathematical result of SUV50% and the volume of the lesions; therefore, the results may be contradicting (e.g., an average or high SUV50% concerning a small area may end up with a small PV50%, which will be translated falsely as a negative lesion). Jung et al., in a volumetric approach of metastatic lesions measured on 11C-MET-PET, reported a different optimal cutoff of T/B values depending on the metabolic tumor volume (MTV) and consequently, the size of the lesion [22]. Unterrainer et al. noted 18F-FET uptake in small untreated brain metastatic lesions of diameter 0.6–1 cm, even though all 18F-FET-negative metastases were < 1 cm, concluding that radiopharmaceutical uptake was not dependent on the lesion size [24]. All the SUV values could significantly discriminate between positive from negative lesions, with T/B cutoff showing the best accuracy. T/B ratio seems to reflect a clearer result of radiopharmaceutical uptake, perhaps due to the more constant background activity and to the lower values of SUVmax and SUVpeak. Nguyen et al. used SUVmean of the pons for background SUV estimation; however, the rationale was to use that area for vascular SUV calculations as well [25]. In our study, we used SUVmean of the background and T/B of 4.21, which resulted in the best sensitivity and accuracy.

Other PET radiopharmaceuticals besides FDG have also been investigated in the setting of brain metastatic disease with promising results. Radiolabeled amino acids are the most common radiopharmaceuticals used for investigating new and recurrent brain metastasis, distinguishing true progression from pseudo progression, visualizing irradiated brain metastatic lesions, and managing patients for long-term treatment [22,23,24,26,27]. Finally, a cost-effectiveness analysis showed that the utilization of 18F-FET, in addition to MRI, for recurrent brain metastasis after radiotherapy may be cost-effective [26]. Apart from amino acids, 11C-choline seems to be taken up by metastatic brain lesions as well [28]. The purpose of our study was to evaluate the performance of 18F-FLT-PET/CT in metastatic brain lesions in terms of sensitivity, specificity, and accuracy regardless of prior treatments and in the evaluation of several SUV and proliferative volumes. Any further analysis according to primary tumor or regarding patient management and prognosis was beyond the scope of this study. Further investigation to that direction may be performed.

Our study has several limitations. It is a retrospective study with a small, non-uniform sample with mixed primary sites and therapies that could interfere with our results. It is unknown if the primary tumor could have an impact on 18F-FLT accumulation in brain metastatic lesions and needs further investigation. Moreover, different prior treatments could be implicated in BBB disruption and 18F-FLT uptake. Another factor that could influence the estimation of the diagnostic accuracy of 18F-FLT-PET is the lack of histologic confirmation as diagnostic standard and the fact that final characterization of the lesions was made based mainly on follow up and MRI. However, our results indicate that FLT can depict metastatic brain lesions and can be used in that field as a complement to MRI.

## 5. Conclusions

Our results indicate that 18F-FLT is accumulated in metastatic lesions, and its sensitivity and accuracy are high. Therefore, it could be used in as a complement to other imaging procedures. T/B and SUVmax provide the best diagnostic accuracy, while PV50% and PV75% are of less diagnostic significance. In the future, FLT may become more widely used for investigation of secondary brain lesions and more robust estimations could be made using this modality.

## Figures and Tables

**Table 1:** Patients’ characteristics: M: Male, F: Female, No.: number, y: years old, CMTx: chemotherapy, RT: radiotreatment in the brain, RT naive: refer to patients who never had undergone radiotreatment in the brain. In one case the patient had undergone surgical excision after radiotherapy and is mentioned separately. Surgical excision refers to all patients who had surgical excision of a brain lesion. Treatment naive refers to patients who had never undergone neither chemotherapy nor radiotreatment. Per examination analysis refers to the number of 18F-FLT-PET/CT examinations.

Characteristics	No.
M/F	9/5
Age (y) (average, range)	64, 40–83
Primary disease	
(Per examination analysis)	
*Lung*	9
*Breast*	7
*Melanoma*	2
*Thymoma*	2
Treatments	
(per examination analysis)	
*CMTx*	11
*RT*	16
*Surgical excision after RT*	1
*RT naive*	3
*Surgical excision*	6
*Treatment naive*	0

**Table 2:** Mean (standard deviation) of different parameters for positive and negative lesions. Comparisons were made with independent *t*-test. *p* < 0.05 is considered statistically significant.

	Mean (SD)		*p*-Value (*t*-Test)
	Negative	Positive	
SUVmax	0.625 (0.48)	1.78 (1.57)	0.003
SUVpeak	0.45 (0.41)	1.17 (1.04)	0.013
T/B	4.55 (2.76)	9.88 (9.25)	0.017
SUV1cm^3^	0.46 (0.41)	1.18 (1.08)	0.014
SUV0.5cm^3^	0.5 (0.43)	1.34 (1.24)	0.009
SUV50%	0.39 (0.3)	1.13 (1.06)	0.004
PV50%	1.85 (3.36)	2.89 (3.75)	0.52
SUV75%	0.52 (0.39)	1.48 (1.33)	0.003
PV75%	0.31 (0.58)	0.56 (0.74)	0.384

**Table 3:** Median (interquartile range) of different parameters for positive and negative lesions. Comparisons were made with a non-parametric Kruskall-Wallis test. *p* < 0.05 is considered statistically significant.

	Median (IQR)		Kruskall-Wallis
	Negative	Positive	*p*-Value
SUVmax	0.49 (0.32–0.6)	1.29 (0.635–2.21)	0.017
SUVpeak	0.345 (0.2–0.42)	0.86 (0.425–1.475)	0.017
T/B	3.665 (2.64–5.27)	6.43 (4.58–11.66)	0.045
SUV1cm^3^	0.35 (0.21–0.43)	0.87 (0.41–1.435)	0.032
SUV0.5cm^3^	0.385 (0.23–0.46)	0.95 (0.47–1.575)	0.018
SUV50%	0.31 (0.19–0.39)	0.77 (0.425–1.3)	0.02
PV50%	0.604 (0.304–0.823)	1.2284 (0.6991–3.85)	0.123
SUV75%	0.41 (0.26–0.53)	1.06 (0.59–1.85)	0.02
PV75%	0.099 (0.036–0.1176)	0.4 (0.09–0.53)	0.112

**Table 4:** ROC curve analysis for each parameter. The higher the area under the curve (AUC) the better the discriminatory power between positive and negative lesions. SUVmax has the best performance (as depicted by the AUC values and confidence intervals). *p* < 0.05 is considered statistically significant.

Test	AUC	95% Confidence Interval	*p*-Value
SUVmax	0.815	0.637–0.993	0.017
SUVpeak	0.815	0.631–0.999	0.017
T/B	0.765	0.568–0.963	0.045
SUV1cm^3^	0.784	0.586–0.982	0.032
SUV0.5cm^3^	0.812	0.625–0.999	0.018
SUV50%	0.809	0.632–0.986	0.02
SUV75%	0.809	0.632–0.986	0.02

**Table 5:** Optimal cut-offs and the corresponding false positive and false negative results, sensitivity (sens), specificity (spec) and accuracy. OC: optimal cutoff. No.: number of lesions, FP: false positive, FN: false negative.

OC	SUVmax ≥ 0.615	SUVpeak ≥ 0.425	T/B ≥ 4.21	SUV1cm^3^ ≥ 0.455	SUV0.5cm^3^ ≥ 0.47	SUV50% ≥ 0.405	SUV75% ≥ 0.535
FP (No.)	1	1	2	1	1	1	1
FN (No.)	6	7	4	8	7	6	6
Sensitivity	77.8%	74.1%	85.2%	70.4%	74.1%	77.8%	77.8%
Specificity	83.3%	83.3%	66.7%	83.3%	83.3%	83.3%	83.3%
Accuracy	78.8%	75.8%	81.8%	72.7%	75.8%	78.8%	78.8%

## References

[1] Crippa F., Alessi A., Serafini G.L. (2012). PET with radiolabeled aminoacid. Quat. J. Nucl. Med. Mol. Imaging.

[2] Tsitsia V., Svolou P., Kapsalaki E., Theodorou K., Vassiou K., Valotassiou V., Georgoulias P., Fezoulidis I., Tsougos I. (2017). Multimodality-multiparametric brain tumors evaluation. Hell. J. Nucl. Med..

[3] Wang W., Hu Y., Lu P., Li Y., Chen Y., Tian M., Yu L. (2014). Evaluation of the diagnostic performance of magnetic resonance spectroscopy in brain tumors: A systematic review and meta-analysis. PLoS ONE.

[4] Mertens K., Slaets D., Lambert B., Acou M., De Vos F., Goethals I. (2010). PET with (18)F-labelled choline-based tracers for tumour imaging: A review of the literature. Eur. J. Nucl. Med. Mol. Imaging.

[5] Quartuccio N., Asselin M.C. (2018). The validation path of hypoxia PET imaging: Focus on brain tumours. Curr. Med. Chem..

[6] Idema A.J., Hoffmann A.L., Boogaarts H.D., Troost E.G., Wesseling P., Heerschap A., van der Graaf W.T., Grotenhuis J.A., Oyen W.J. (2012). 3′-Deoxy-3′-18F-fluorothymidine PET-derived proliferative volume predicts overall survival in high-grade glioma patients. J. Nucl. Med..

[7] Nikaki A., Angelidis G., Efthimiadou R., Tsougos I., Valotassiou V., Fountas K., Prasopoulos V., Georgoulias P. (2017). 18F-fluorothymidine PET imaging in gliomas: An update. Ann. Nucl. Med..

[8] Muzi M., Spence A.M., O’Sullivan F., Mankoff D.A., Wells J.M., Grierson J.R., Link J.M., Krohn K.A. (2006). Kinetic analysis of 3′-deoxy-3′-18F-fluorothymidine in patients with gliomas. J. Nucl. Med..

[9] Shinomiya A., Kawai N., Okada M., Miyake K., Nakamura T., Kushida Y., Haba R., Kudomi N., Yamamoto Y., Tokuda M. (2013). Evaluation of 3′-deoxy-3′-[18F]-fluorothymidine (18F-FLT) kinetics correlated with thymidine kinase-1 expression and cell proliferation in newly diagnosed gliomas. Eur. J. Nucl. Med. Mol. Imaging.

[10] Ferdová E., Ferda J., Baxa J., Tupý R., Mraček J., Topolčan O., Hes O. (2015). Assessment of grading in newly-diagnosed glioma using 18F-fluorothymidine PET/CT. Anticancer Res..

[11] Yamamoto Y., Ono Y., Aga F., Kawai N., Kudomi N., Nishiyama Y. (2012). Correlation of 18F-FLT uptake with tumor grade and Ki-67 immunohistochemistry in patients with newly diagnosed and recurrent gliomas. J. Nucl. Med..

[12] Jeong S.Y., Lee T.H., Rhee C.H., Cho A.R., Il Kim B., Cheon G.J., Choi C.W., Lim S.M. (2010). 3′-Deoxy-3′-[(18)F]fluorothymidine and O-(2-[(18)F]fluoroethyl)-L-tyrosine PET in patients with suspicious recurrence of glioma after multimodal treatment: Initial results of a retrospective comparative study. Nucl. Med. Mol. Imaging.

[13] Chalkidou A., Landau D.B., Odell E.W., Cornelius V.R., O’Doherty M.J., Marsden P.K. (2012). Correlation between Ki-67 immunohistochemistry and 18F-fluorothymidine uptake in patients with cancer: A systematic review and meta-analysis. Eur. J. Cancer.

[14] Chen W., Delaloye S., Silverman D.H., Geist C., Czernin J., Sayre J., Satyamurthy N., Pope W., Lai A., Phelps M.E. (2007). Predicting treatment response of malignant gliomas to bevacizumab and irinotecan by imaging proliferation with [18F] fluorothymidine positron emission tomography: A pilot study. J. Clin. Oncol..

[15] Peat J., Burton B. (2005). Categorical and continuous variables: Diagnostic statistics. Medical Statistics: A Guide to Data Analysis and Critical Appraisal.

[16] O’Sullivan C.C., Lindenberg M., Bryla C., Patronas N., Peer C.J., Amiri-Kordestani L., Davarpanah N., Gonzalez E.M., Burotto M., Choyke P. (2016). ANG1005 for breast cancer brain metastases: Correlation between 18F-FLT-PET after first cycle and MRI in response assessment. Breast Cancer Res. Treat..

[17] Hoshikawa H., Kishino T., Mori T., Nishiyama Y., Yamamoto Y., Mori N. (2013). The value of 18F-FLT PET for detecting second primary cancers and distant metastases in head and neck cancer patients. Clin. Nucl. Med..

[18] Dittmann H., Dohmen B.M., Paulsen F., Eichhorn K., Eschmann S.M., Horger M., Wehrmann M., Machulla H.J., Bares R. (2003). [18F]FLT PET for diagnosis and staging of thoracic tumours. Eur. J. Nucl. Med. Mol. Imaging.

[19] van der Hiel B., Haanen J.B.A.G., Stokkel M.P.M., Peeper D.S., Jimenez C.R., Beijnen J.H., van de Wiel B.A., Boellaard R., van den Eertwegh A.J.M., REPOSIT study group (2017). Vemurafenib plus cobimetinib in unresectable stage IIIc or stage IV melanoma: Response monitoring and resistance prediction with positron emission tomography and tumor characteristics (REPOSIT): Study protocol of a phase II, open-label, multicenter study. BMC Cancer.

[20] Tixier F., Vriens D., Cheze-Le Rest C., Hatt M., Disselhorst J.A., Oyen W.J., de Geus-Oei L.F., Visser E.P., Visvikis D. (2016). Comparison of tumor uptake heterogeneity characterization between static and parametric 18F-FDG PET images in non-small cell lung cancer. J. Nucl. Med..

[21] Wahl R.L., Jacene H., Kasamon Y., Lodge M.A. (2009). From RECIST to PERCIST: Evolving considerations for PET response criteria in solid tumors. J. Nucl. Med..

[22] Jung T.Y., Kim I.Y., Lim S.H., Park K.S., Kim D.Y., Jung S., Moon K.S., Jang W.Y., Kang S.R., Cho S.G. (2017). Optimization of diagnostic performance for differentiation of recurrence from radiation necrosis in patients with metastatic brain tumors using tumor volume-corrected 11C-methionine uptake. EJNMMI Res..

[23] Yomo S., Oguchi K. (2017). Prospective study of 11C-methionine PET for distinguishing between recurrent brain metastases and radiation necrosis: limitations of diagnostic accuracy and long-term results of salvage treatment. BMC Cancer.

[24] Unterrainer M., Galldiks N., Suchorska B., Kowalew L.C., Wenter V., Schmid-Tannwald C., Niyazi M., Bartenstein P., Langen K.J., Albert N.L. (2017). 18F-FET PET uptake characteristics in patients with newly diagnosed and untreated brain metastasis. J. Nucl. Med..

[25] Nguyen N.C., Yee M.K., Tuchayi A.M., Kirkwood J.M., Tawbi H., Mountz J.M. (2018). Targeted therapy and immunotherapy response assessment with F-18 Fluorothymidine positron-emission tomography/magnetic resonance imaging in melanoma brain metastasis: A pilot study. Front. Oncol..

[26] Heinzel A., Müller D., Yekta-Michael S.S., Ceccon G., Langen K.J., Mottaghy F.M., Wiesmann M., Kocher M., Hattingen E., Galldiks N. (2017). O-(2-18F-fluoroethyl)-L-tyrosine PET for evaluation of brain metastasis recurrence after radiotherapy: An effectiveness and cost-effectiveness analysis. Neuro Oncol..

[27] Romagna A., Unterrainer M., Schmid-Tannwald C., Brendel M., Tonn J.C., Nachbichler S.B., Muacevic A., Bartenstein P., Kreth F.W., Albert N.L. (2016). Suspected recurrence of brain metastases after focused high dose radiotherapy: Can [18F]FET-PET overcome diagnostic uncertainties?. Radiat. Oncol..

[28] Rottenburger C., Hentschel M., Kelly T., Trippel M., Brink I., Reithmeier T., Meyer P.T., Nikkhah G. (2011). Comparison of C-11 methionine and C-11 choline for PET imaging of brain metastases: A prospective pilot study. Clin. Nucl. Med..

